# Where Is Medical Practice In India Heading?

**DOI:** 10.4103/0973-1229.27605

**Published:** 2006

**Authors:** Sunil K. Pandya

**Affiliations:** * *Department of Neurosurgery, Jaslok Hospital and Research Centre, Dr. G. V. Deshmukh Marg, Mumbai 400026. Email: shunil@vsnl.com*

**Keywords:** *Medical Education*, *Medical Practice*, *Medical Ethics*

## Abstract

Medical practice is based on teaching, learning and examples set by seniors. Past and present practices are briefly analysed. Current trends do not justify optimism. The poor patient is likely to be sidelined as doctors reach out to the rich and powerful in this country and those bringing in American dollars from abroad. While corrective steps are possible, it is unlikely that they will be implemented.

## Introduction

The great teachers of medicine have consistently taught the same lessons. Medicine and medical practitioners exist to cure diseases when possible; relieve symptoms to the extent enabled by their talents and expertise; and comfort patient and family, always. We are exhorted to put the interests of the patient and family above all else. Treatment must always follow diagnosis and must use the most efficacious and least expensive means.

Summing up these teachings, we were told to follow the golden rule – do unto your patient, as you would expect other physicians to do unto you, were you the patient.

Viewing the present medical practices in India fills the observer with a sense of dismay. In most physicians the urge to help others has been replaced by a compelling drive to gain immense wealth in the shortest possible period. As a senior teacher put it recently, medicine is no more a calling or a vocation. It has become just another form of commerce.

In order to understand the directions in which Indian medical practice is headed it is important to review past and current practices.

## Teaching, Past and Present

I was fortunate in my teachers. They were learned and wanted to impart whatever they had learnt. A few were flamboyant but benignly so. The most striking example of the latter is Dr. O. P. Kapoor, who always arrived at the J. J. Hospital impeccably dressed, in a large car driven by a smartly uniformed chauffeur. He held classes for his students at the Grant Medical College and in his consulting rooms after his office hours, often starting around 10 p.m. and continuing into the small hours. Tea and snacks were served to the students at his expense. He continues his teaching now in Birla Matushri Sabhagraha, Mumbai, a hall that gets packed to capacity. He has never charged a paisa* for his efforts.

Contrast this with the current scene in many medical colleges. Full-time Professors and Associate Professors are often not to be found on the campus during office hours, as they are busy treating patients in large private hospitals. The licence to practice *after* office hours granted by the powers that be in the Municipal Corporation of Greater Mumbai has been cheerfully modified to practice at *any* hour. Since the Municipal Corporation, by its own admission, was unable to monitor and check full-time staff members indulging in private practice before the recent sanction, it will turn a blind eye to the present misdemeanour as well. Students - undergraduate and postgraduate - and patients alike, will suffer as a consequence.

The ills of coaching classes in medicine, fuelled by large sums paid by students to the teachers and facilitated by leakages of questions to be asked at forthcoming examinations, have been amply documented and are public knowledge.

In sharp contrast were my teachers. Many of them were widely read. They discussed art, literature, history (including the history of medicine) and philosophy even as they taught us clinical medicine at the bedside. Dr. Rustom N. Cooper opened up his personal library containing hundreds of books on the history of medicine to an unknown medical student who humbly approached him for a particular volume. Dr. Rustom Jal Vakil published his own essays on the history of medicine even as he carried out his research on *Rauwolfia Serpentina* that was to win him the *Lasker Award*. He also inspired at least two of his students to study the history of their *alma mater* - the Grant Medical College and Sir J. J. Group of Hospitals. Dr. P. K. Sen, striding the corridors of the K. E. M. Hospital, was as much at home in Bengali literature, painting in oils, and elocution, as in cardiac surgery. Dr. R. A. Bhalerao, his student and disciple, is well known not only for his work on surgical treatment of diseases of the liver but also for his contributions to Marathi drama. Dr. Farokh Udwadia has gained eminence not only as a gentleman-physician but also as a medical historian and a musician.

I tried to list the extracurricular activities of leading teachers in medical colleges in Mumbai today. Barring a few exceptions, I find little to admire.

What does this portend? What can we expect of students thus taught and inspired?

If present trends continue and teachers prefer the acquisition of personal wealth over the stimulation of young minds to learn, ponder, question and innovate, the already low standards of medical education will, inevitably, fall further. The ill consequences will be progressively evident when the students of today become the teachers, and practitioners, of tomorrow.

## Practice, Now And In the Past

Using expensive laboratory or imaging tests in preference to clinical acumen in the making of a diagnosis is becoming all-too-evident. Attempts at understanding the natural history of the patient’s illness is giving way to rapid-fire investigations and gunshot therapy.

In part the use of expensive tests may also be gaining prominence because of the ‘incentives’ offered by laboratories and scan centres. A portion of the fees paid by the patient to the centre is passed on to the clinician. This unethical act is camouflaged by the use of terms such as ‘fees for clinical assessment’ or ‘provision of clinical details’. Such sugar-coating appears to assuage the conscience of many colleagues.

As an impressionable medical student, I learnt about physicians such as Dr. Minocher Mody. Let me quote just one of the lessons I imbibed from his practice. At the height of his career, with a waiting room overflowing with patients, he received a telephone call from a family physician.

The conversation was as follows:

*Family physician*: Sir, I would like you to spare some time today to come with me to see a seriously ill patient. I have done all I could but have not been able to help him.

*Dr. Mody*: I would like to help but have several patients waiting to see me. Could you, please, request another consultant to see the patient with you instead? If you wish, I can recommend Dr. ABC, whose opinion and advice I respect.

*Family physician*: Thank you. I will try and get him to see this patient.

A few minutes later the telephone rang again. It was the same physician.

*Family physician*: I’m sorry to trouble you sir, but Dr. ABC cannot see this patient. I have also tried Dr. XYZ but find him busy too. I am quite worried about my patient.

*Dr. Mody*: Why is it that none of these physicians can see your patient?

*Family physician*: I’m afraid I do not know the answer. I had to tell them that the patient is a poor mill-worker and lives in a chawl*. It may be that the doctors are apprehensive about whether they will be paid their fees.

*Dr. Mody*: So the patient is poor. Why did you not tell me this earlier? Let me see… Is it possible for you to be at my rooms in an hour? I usually have my lunch then. I will come with you instead and see your patient.

Dr. Mody saw the patient, spending half an hour with him. As he left, instead of asking for a fee, he slipped several currency notes under the patient’s pillow, explaining to the patient’s family that they would need them for the medicines he had prescribed. Dr. Mody skipped his lunch that day. He resumed examination of the patients in his waiting room as soon as he returned to his clinic.

Dr. Homi Shapurji Mehta taught us forensic medicine and medical jurisprudence. His eminence as a Police Surgeon was such that senior judges listened to him with respect when he gave evidence, or opined as an expert on medico-legal matters. His talks on medical ethics and medical etiquette left a deep impression on our minds. I recall his insistence that commercialisation of medicine lowered the dignity of the profession - a profession he held sacred – and was therefore to be strictly avoided. I also recall his teaching us that when the needs of the patient demanded obtaining a consultation with another doctor, personal vanity had to be set aside so that the best available doctor was called in to benefit the patient. His suggestions on how second opinion was to be obtained, and given, remain invaluable. He was unsparing in his criticism of the practice of splitting fees. ‘The evil of dichotomy cannot be over-emphasised. It destroys the good and unselfish doctor-patient relationship.’ It is not surprising that Dr. Mehta dedicated his book *Medical law and ethics in India* to ‘…Dr. Rustomji Nusserwanji Cooper …in fond appreciation of his outstanding qualities as a surgeon and his inspiring and ennobling nature’.

I served as a house surgeon to Dr. Hiralal K. Doctor. Seemingly gruff and at times crude in his language, he had a heart of gold. On the first day of each month he would enter the ward and hand over an envelope to the sister in charge. He uttered just one sentence: ‘If you need any more let me know.’ He proceeded around the ward as usual before departing. Intrigued, I asked Sister what was in the envelope. She opened it and exposed the contents - several currency notes, each of a hundred rupees. ‘I have standing instructions to use these for poor patients under Dr. Doctor’s care. He never asks me for accounts, nor does he wish to know who benefits from them.’

Dr. W. D. Sulakhe and Dr. P. D. Anjaria, honorary professors of medicine at J. J. Hospital and K. E. M. Hospital respectively, travelled to and from their institutions by bus throughout their careers. They did not find this beneath their dignity.

It is salutary to wonder what these physicians would think of current mores.

Our young graduates now see teachers, supposedly in full-time positions, rush to private hospitals during office hours to treat patients there, in preference to those under their care in teaching hospitals. They see patients being referred to other consultants not because the latter are the best in the field or most suited to treating the patient, but because of high incentives offered by them to the referring physician. They see doctors who have flouted all norms of ethical practice rolling around in Mercedes cars and flaunting their connections with political powers and film stars.

Such examples set by teachers influence impressionable minds. If our doctors of the future are to follow these examples – as well they might - the plight of the poor and middle class patients can only be imagined. As it is, these patients receive shoddy treatment at most centres. When power and wealth become the goal of the average physician, the poor will find no place in the minds or hearts of their physicians.

We are also witness to a scramble to attract patients from abroad. Five-star hospitals and private consultants advertise their wares and offer inducements. One example of the latter is a holiday for the patient and companion in Goa or Kerala, the cost of which is included in the medical bill. Such enterprise would be laudable had we already ensured treatment for the humblest and poorest Indian. Unfortunately this is a goal we can only dream of at present. Under the circumstances, the advertisers show themselves up as crass opportunists out to make fast dollars with little concern for their poorer countrymen.

## Medical Meetings And Conferences, Then And Now

When I graduated in medicine and was a resident doctor, our teachers were primarily academicians. They thirsted for understanding of the natural history of diseases peculiar to India and studied them earnestly. As a consequence, Drs. Noshir Wadia, Gajendra Sinh, Jaggubhai G. Parikh, M. M. Wagle, Homi M. Dastur and others published their findings in papers that remain classics in Indian medical literature.

When they, and like-minded teachers, organised medical conferences, they chose medical colleges as venues and provided rich fare for the intellect. Whilst gastric needs were not ignored, they were given second place. Inexpensive and simple business lunches were the order of the day. Postgraduate students and junior teachers could easily afford the registration fees for these conferences. Those attending the meetings did so to learn. A respected teacher inaugurated the conference. The presence of pharmaceutical companies and manufacturers of instruments was felt only in the exhibition hall where they displayed their wares in modest stalls. They did not intrude on the conference.

Conferences are now held in starred hotels. Lavish meals are the rule. Delegates cannot escape the all-pervading presence of companies making drugs and instruments. Slides advertising products are projected between two successive talks. We are constantly being told which company has sponsored breakfast, lunch and dinner. At one meeting, the biscuits served at tea were embossed with the brand name of the drug being sold by the company paying for them. Senior consultants and their families are flown in and housed in starred hotels by companies. They have chauffeured cars at their disposal all the time. Small wonder that whilst the dining halls are crowded all the time, the lecture halls show a progressively diminishing population as we proceed from the inauguration of the conference to the last day. The inauguration is performed by a powerful politician and is often delayed till this ‘dignitary’ arrives. The sycophancy of senior conference officials at his arrival sickens the pensive observer.

Since up-and-coming doctors attend these meetings, the effects on them of current mores cannot but further demean the professed goals of such conferences. They are quick to learn that to rise in the hierarchy of officialdom of societies or associations, it is necessary to cultivate those in power and that commerce must dominate over academic excellence. The humble researcher is consigned to oblivion. The flashy star performer serves as the model on which they groom themselves.

## Interaction With Manufacturers Of Drugs, Instruments And Other Accessories

Interaction with manufacturers of drugs, instruments and other accessories in the past, were based on the need for the development of a new instrument devised by a surgeon, or the development of a facility beneficial to patients but hitherto unavailable in the city. The discussions between doctors and industrialists were academic. The inventor and the manufacturer equally shared the profit, if any, from the newly developed instrument. In several instances, the doctor’s share of the profit was used for the benefit of poor patients at the teaching hospital where he served as a consultant.

There has been a dramatic shift in values. The relations between some senior members of the profession and manufacturers exclude the welfare of the patient. Blatant demands by doctors for first-class air tickets for themselves and their families to distant destinations, accommodation in five star hotels and other such ‘facilities’ are linked to threats of ‘or else…’ When the doctor has a huge practice, heads a large institution purchasing goods worth crores annually, or is an influential office-bearer in a national medical association, most company managers give in. All this, of course, is at the expense of the patients who end up paying higher prices.

Institutions lacking clean toilets, freshly laundered bed sheets and pillowcases, and blankets without gaping holes, cheerfully spend huge sums on the latest Computerised Tomographic Scanner or Magnetic Resonance Imaging machine.

Private hospitals purchase equipment costing millions of U. S. dollars. An example is the ‘Whole Body Gamma Knife’ used for highly intensive radiotherapy in tumours. When the returns do not match the huge capital expenditure, pressure is exerted on clinicians to refer greater numbers of patients for such treatment, the indications being broadened to the point of absurdity.

Since huge international companies must, of necessity, keep bringing out newer and ever-more-expensive machines to maintain their profit margins, they will exert increasing pressure on clinicians and other doctors. Corrupt administrators of hospitals purchasing expensive machines, benefiting from the percentages of costs handed to them by the manufacturers, also exert such pressure.

Will the clinicians of the future resist the purchase of machines that are not cost-effective? Will they insist that the purchase of any new machine is justified only when it is significantly better than the one in use and will lower costs to the patient?

My experience does not permit optimism.

## Medical Ethics

Were we faithful to ethical principles, medical practice in India would have been truly beneficial to patients. Why have so many of us strayed from the straight and narrow path?

The teaching of medical ethics appears to have fallen by the wayside. During the courses on ethics conducted by us at teaching institutes all over India we have met with a common question: ‘Why is it that the topics discussed at this course have never been featured during our entire undergraduate and postgraduate training?’

There are few teachers who can inspire impressionable medical students and young residents. Our youngsters are no fools. They are able to separate pious verbal outpourings by their seniors from their practices intended to extract the last rupee from each patient and glorify themselves. The preaching of courtesy and discourses on the rights of patients are followed by rude and rough treatment of the poor patient and fawning over the rich and powerful. The teacher forcing his juniors to include his name as the first author of papers for publication when he has done none of the work indicates that ‘positional’ might is right.

## Corrective Steps

The principles and practice of medical ethics must be one of the cornerstones of medical education.

Mere preaching will not work. Teachers and senior practitioners must set examples of rational, scientific and selfless treatment of patients. The patient must form the centre of attention, every act being performed with his welfare in mind. As costs escalate and widespread poverty persists, all efforts must be made at providing the best medical care at the least expense.

## What Does the Future Hold?

We are witnessing a great effort at fostering ‘medical tourism’. Private hospitals have special departments of ‘public relations’ - a euphemism for efforts at attracting increasing numbers of well-to-do patients to their hospitals.

Public and private hospitals - at least in the city of Mumbai - have turned a blind eye to inconvenient rules and regulations. I have already referred above to full-time professors in teaching hospitals today who blatantly practice in private hospitals during office hours.

*Indian Journal of Medical Ethics (http://www.ijme.in/)*- the only journal on the subject in the country - has to struggle to survive.

Resident doctors in teaching hospitals continue to paralyse them by going on strike every few years, ostensibly to improve conditions for patients, their own security, or their self-respect. In doing so they cause untold harm to poor patients. Each time, the strike is called off after haggling over raises in their stipends. Resident doctors and administrators in the state government have, by now, perfected their respective stances and ensure that the strikes are called off within a week or ten days. They return to their respective tasks with satisfaction at a skirmish well fought. I doubt greatly whether either the doctors or the administrators spare thought, concern or regret at the irreparable harm done to innocent patients who have nothing whatsoever to do with the purported reasons for the strike.

Role models of the kind I had are now rare.

I would very much have liked to sound notes of optimism. In all honesty, I cannot do so.

## Concluding Remarks

The Indian newspapers are full of optimism on the rising stock market indices, strides taken by our major commercial organisations, the takeovers of foreign companies by Indian enterprises and Indians abroad who are listed amongst the world’s richest persons.

Major Indian private hospitals, especially those forming chains promoted by commercial houses, tout their commercial successes and revel in the latest in medical gadgetry to be found in their departments.

These would have justified pride except for a dilemma posed by a thought enunciated by the Mahatma:

Recall the face of the poorest and the weakest man whom you may have seen and ask yourself if the step you contemplate is going to be of any use to him.

When we apply this test, pride is replaced by humility and a sense of dismay. There is so much to be done for the poorest and the weakest. Are our best minds and our finest medical organisations up to this task? Have they started work on it? Have they even considered this task worthy of them?

## Questions That This Paper Raises

1. Do you agree with the author and share his pessimism? If you do not, give examples of success stories in recent Indian medical education and practice benefiting poor patients.
2. The author does not consider the steps to be taken by the authorities. Can the Government, Municipal Corporations, Associations of doctors, Medical Councils improve matters? If so, what steps do you suggest?
3. What are your experiences of ethics in medical education and practice?
4. There is an argument oft used by many: ‘Everyone in India is corrupt. Name one profession where there is no corruption. Why should anyone expect the medical professionals to remain free from corruption? This is the only way to survive in this country.’ Do you agree?
5. What are your views on the matter of seed and soil? Since medical colleges get as students fiercely competitive individuals who have fought tooth and nail to get in by means that are not necessarily above board, how can you expect the finished products of medical colleges to be angelic doctors?
6. What is wrong in encouraging medical tourism? It will bring in large quantities of wealth from Western and Arab nations and this must, inevitably, benefit the country as a whole.

## About the Author

***Dr. Sunilkumar Krishnalal Pandya**, eminent Neurosurgeon and thinker on medical ethics, joined the Grant Medical College in 1957 and trained at the Sir J.J. Group of Hospitals, Mumbai. He obtained the M.B.B.S. (1961) and M.S. (1965). He joined Dr. Homi Dastur at the Department of Neurosurgery, Seth G. S. Medical College and K. E. M. Hospital in 1967 as a Pool Officer and was appointed to the staff as Assistant Neurosurgeon in 1968. In 1975, on Dr. Dastur's retirement, he was appointed Professor of Neurosurgery. He retired on superannuating in 1998 and has, since, worked at the Jaslok Hospital and Research Centre, Mumbai. He is Editor Emeritus*, IJME (Indian Journal of Medical Ethics); *Journal Ombudsman*, JPGM (Journal of Postgraduate Medicine); *and Member, International Editorial Advisory Board of the* Mens Sana Monographs.

## Footnotes

* Paisa means penny (eds).

* Chawl means a shanty (eds).

